# Laparoscopic versus open pancreatic resection for ductal adenocarcinoma: separate propensity score matching analyses of distal pancreatectomy and pancreaticoduodenectomy

**DOI:** 10.1186/s12885-021-08117-8

**Published:** 2021-04-09

**Authors:** Ke Chen, Yu Pan, Chao-jie Huang, Qi-long Chen, Ren-chao Zhang, Miao-zun Zhang, Guan-yu Wang, Xian-fa Wang, Yi-ping Mou, Jia-fei Yan

**Affiliations:** 1 Department of Hepatopancreatobiliary Surgery, Sir Run Run Shaw Hospital, School of Medicine, Zhejiang University, 3 East Qingchun Road, Hangzhou, 310016 Zhejiang Province China; 2 Department of Gastrointestinal and Pancreatic Surgery, Zhejiang Provincial People’s Hospital, 158 Shangtang Road, Hangzhou, 310014 Zhejiang Province China; 3 Department of Hepatopancreatobiliary Surgery, Ningbo Medical Center, Lihuili Hospital, Ningbo, 315100 Zhejiang Province China

**Keywords:** Laparoscopy, Pancreatectomy, Adenocarcinoma, Morbidity, Survival

## Abstract

**Background:**

Pancreatic ductal adenocarcinoma (PDAC) is a leading causes of cancer mortality worldwide. Currently, laparoscopic pancreatic resection (LPR) is extensively applied to treat benign and low-grade diseases related to the pancreas. The viability and safety of LPR for PDAC needs to be understood better. Laparoscopic distal pancreatectomy (LDP) and pancreaticoduodenectomy (LPD) are the two main surgical approaches for PDAC. We performed separate propensity score matching (PSM) analyses to assess the surgical and oncological outcomes of LPR for PDAC by comparing LDP with open distal pancreatectomy (ODP) as well as LPD with open pancreaticoduodenectomy (OPD).

**Methods:**

We assessed the data of patients who underwent distal pancreatectomy (DP) and pancreaticoduodenectomy (PD) for PDAC between January 2004 and February 2020 at our hospital. A one-to-one PSM was applied to prevent selection bias by accounting for factors such as age, sex, body mass index, and tumour size. The DP group included 86 LDP patients and 86 ODP patients, whereas the PD group included 101 LPD patients and 101 OPD patients. Baseline characteristics, intraoperative effects, postoperative recovery, and survival outcomes were compared.

**Results:**

Compared to ODP, LDP was associated with shorter operative time, lesser blood loss, and similar overall morbidity. Of the 101 patients who underwent LPD, 10 patients (9.9%) required conversion to laparotomy. The short-term surgical advantage of LPD is not as apparent as that of LDP due to conversions. Compared with OPD, LPD was associated with longer operative time, lesser blood loss, and similar overall morbidity. For oncological and survival outcomes, there were no significant differences in tumour size, R0 resection rate, and tumour stage in both the DP and PD subgroups. However, laparoscopic procedures appear to have an advantage over open surgery in terms of retrieved lymph nodes (DP subgroup: 14.4 ± 5.2 vs. 11.7 ± 5.1, *p* = 0.03; PD subgroup 21.9 ± 6.6 vs. 18.9 ± 5.4, *p* = 0.07). These two groups did not show a significant difference in the pattern of recurrence and overall survival rate.

**Conclusions:**

Laparoscopic DP and PD are feasible and oncologically safe procedures for PDAC, with similar postoperative outcomes and long-term survival among patients who underwent open surgery.

## Background

Pancreatic duct adenocarcinoma (PDAC) is currently the fourth leading cause of cancer-related deaths in developed countries and may rank second by the year 2030 [1, 2]. Surgical resection is considered the only method to radically cure this type of cancer [3]. The surgical extent depends on the tumour location: left-sided PDAC should be treated by distal or subtotal pancreatectomy (DP), and PDAC on the pancreatic head should be seeking pancreaticoduodenectomy (PD). Minimally invasive surgery (MIS), which is characterised by laparoscopic interventions, has become the standard of care for many surgical procedures across different specialities. The selection of MIS is the professional objective of surgeons and the most acceptable treatment for patients [4]. Regarding pancreatectomy, while the safety and effectiveness of laparoscopic DP (LDP) has been gradually evaluated [5], laparoscopic PD (LPD) for PDAC is still in its infancy due to the complexity of the operation and the steep learning curve required for its introduction [6, 7]. The Miami International Evidence-based Guidelines suggested that LPD should be exclusive to experienced surgeons in high-volume centres [8]. Additionally, to date, data on laparoscopic pancreatic resection (LPR) for PDAC and oncological outcomes are limited. The varying preferences between surgeons, difficulties in the intracorporeal hand-sewn technique, and expected cumulative and standardised outcomes are challenges in conducting LPR for PDAC [9]. In other words, the therapeutic role of LPR for PDAC has not yet been established [10, 11]. Considering the different natures of LDP and LPD, it is reasonable to separately analyse LDP and LPD for the treatment of PDAC. We first proposed LDP as early as 2003 [12] and conducted LPD for the treatment of PDAC in 2012 after extensive laparoscopic experience [13, 14]. In this study, we evaluated the safety and effectiveness of LDP and LPD by separately comparing their short- and long-term clinical outcomes with those of open DP (ODP) and PD (OPD).

## Methods

### Study design and enrolled patients

The study protocol was approved by the Institutional Review Board of Zhejiang University. Written consent was obtained from each patient prior to surgery. Patients diagnosed with PDAC between January 2004 and February 2020 were identified from a prospectively maintained pancreatic database. The same surgical team with extensive laparoscopic experience performed all LPR procedures in all patients included in this study [15–19]. The diagnosis of PDAC was based primarily on preoperative imaging, specifically abdominal computed tomography or magnetic resonance imaging. All of the included cases met the resectable criteria laid down by the National Comprehensive Cancer Network guidelines for preoperative assessments. Surgical procedures for PDAC included DP and PD. Multidisciplinary treatment (MDT) was routinely conducted for pancreatic surgery during which the decision of surgical approaches was made, followed by a presentation of the surgical approach to patients and their families. Patients who previously underwent palliative resection or total pancreatectomy for PDAC and those who had distant metastasis were excluded from this study. Prior abdominal surgery was not considered a contraindication to laparoscopic surgery.

**Fig. 1:**
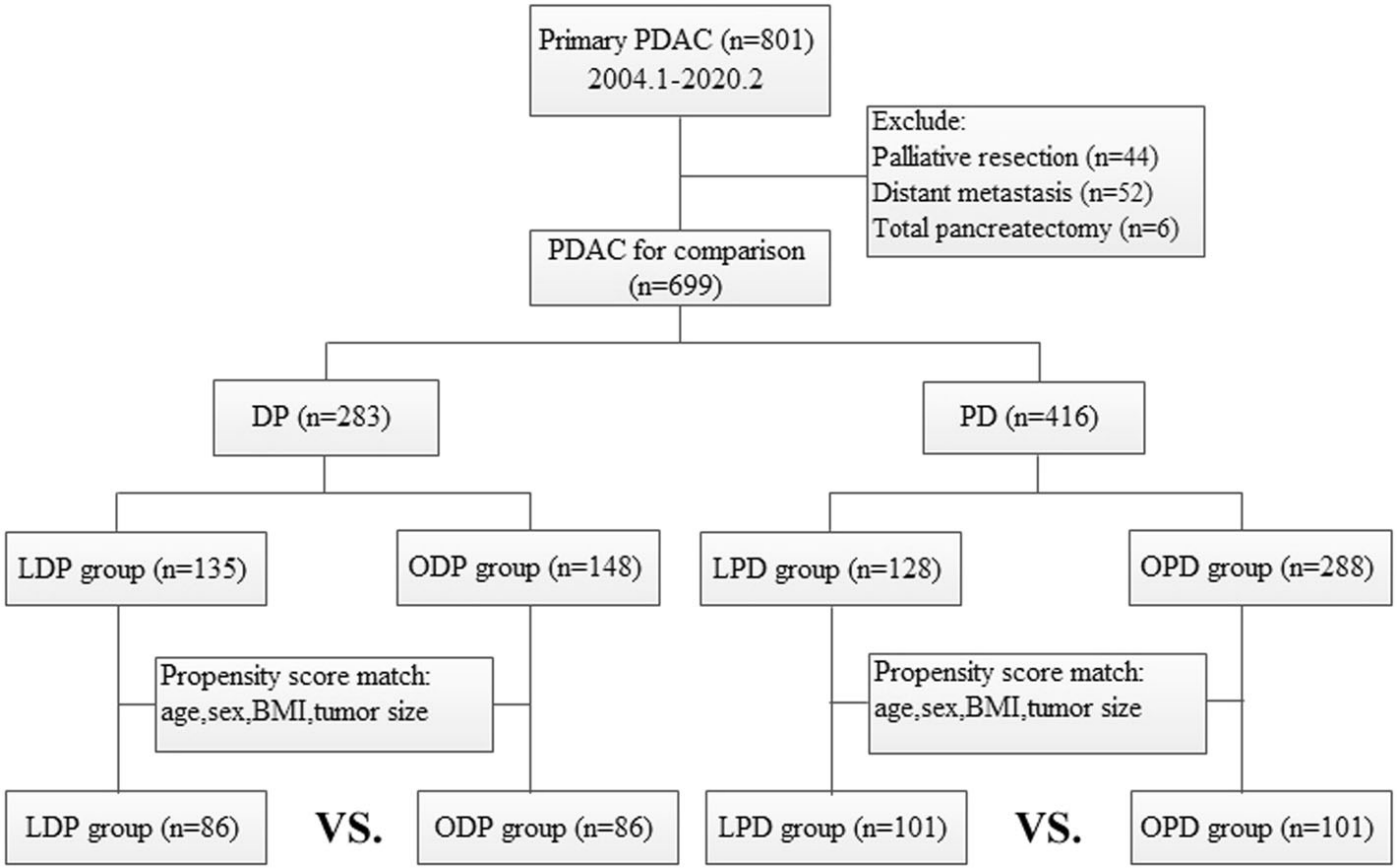
Flow chart of patient selection and literature search strategy

The DP group patients were divided into two subgroups: those undergoing LDP and those undergoing ODP. To minimise the effect of confounding factors and potential bias between the LDP and ODP groups, 1:1 propensity score matching (PSM) was performed via logistic regression analysis. Variables included in the matching model were age, sex, body mass index (BMI), and tumour size. Patients in the PD group were divided in the same way and similar statistical analysis was performed. A flow chart of patient selection is shown in Fig. 1. Patients were evaluated in an intention-to-treat (ITT) manner. Data on patient demographics, clinical presentation, surgical outcomes, tumour characteristics, lymph node status, resection margins, and long-term oncologic outcomes were compared. Because there was a relatively high conversion rate in the LPD subgroup, we also compared the effect of conversion to laparotomy with that of complete resection under laparoscopy to assess the impact of conversion.

### Definitions and data collection

Patients’ demographic factors included age, sex, BMI, American Society of Anesthesiologists (ASA) score, co-morbidity, previous abdominal surgery history, preoperative total bilirubin, and CA19–9. Perioperative outcomes were evaluated in terms of the operative time, bleeding, transfusion, hospital stay, complications, adjuvant treatment, and time interval to adjuvant treatment. Postoperative pancreatic fistula (POPF) [20], delayed gastric emptying (DGE) [21], and post-pancreatectomy haemorrhage (PPH) [22] were defined and classified according to the criteria set out by the International Study Group of Pancreatic Surgery. Complications were evaluated based on the Clavien–Dindo classification system [23], in which grades I and II were grouped as minor, and grades III–V were grouped as major complications. Adjuvant treatment involved either postoperative chemotherapy (e.g., gemcitabine, S-1) or chemoradiotherapy (e.g., gemcitabine plus radiotherapy). Clinicopathologic factors included cell differentiation, tumour size, R status, total number of lymph nodes (LNs), vascular and perineural invasion, T stage, and N stage. R0 resection was defined as no microscopic invasion at the surgical resection margin. TNM stage was classified according to the American Joint Committee on Cancer staging system (8th edition) [24].

Tumour recurrence was classified as either locoregional, extra-pancreatic, or multiple. Tumours in the surrounding organs, pancreatic remnants, or locoregional LNs could show locoregional recurrences. Peritoneal, distant lymphatic, or haematogenous metastases can be involved in extrapancreatic recurrence. Tumour recurrence is evidenced by the intraabdominal soft tissue surrounding the operative site and/or distant metastases. Disease-free survival (DFS) refers to the time between operation and the diagnosis of recurrence or censoring. Overall survival (OS) is defined as the time between operation and fatality.

### Operative procedures

The operation has been detailed previously [15–17]. For the surgeon and the assistant, there were five ports inserted. The surgical extension and protocol were identical to those for open surgeries. Standard lymphadenectomy was performed in LDP, including at least LN located in the hilum of the spleen (No. 10), along the splenic artery (No. 11), and along the internal border of the body and tail of the pancreas (No. 18). Tumours of the pancreatic body also included the LN located around the celiac axis [25]. Dissection was primarily conducted from the right to the left. The splenic artery and vein were separated at the root. The soft tissue surrounding the common hepatic artery and celiac trunk were dissected in a “medial-to-lateral” manner. Additionally, the distal pancreas and the spleen were removed. The LN stations included in the lymphadenectomy for LPD were 5 (suprapyloric), 6 (infrapyloric), 8a (common hepatic artery), 12b-c (along the bile duct and cystic duct), 13a-b (along the head of the pancreas), 14a-b (along the right lateral side of the superior mesenteric artery), and 17a-b (along the anterior face of the head of the pancreas). The entire retroperitoneal soft tissue was removed. For the reconstruction based on the same principles of in pancreaticojejunostomy (PJ), the intracorporeal Child’s approach was adopted. An end-to-side PJ was performed when the largest diameter of 2 mm was reached at the pancreatic duct even though it was difficult to identify. In comparison, duct-to-mucosa PJ is applicable when the pancreatic ducts exceed 2 mm in diameter. All samples and their margins were subjected to intraoperative frozen section examinations.

### Statistical analysis

All statistical analyses were conducted using the SPSS software (Version 23.0, IBM Corp., Armonk, NY) to treat the population, which means intervention was provided for all patients. When the distribution was considered normal, continuous variables were denoted as mean and standard deviation (SD). Otherwise, the median was used. Categorical variables are indicated by absolute numbers and percentages. To compare continuous variables, the Student’s t-test or the Mann–Whitney U test performed, as appropriate. Additionally, based on the functions provided in the software, the Chi-square test or the Fisher’s exact test was performed for categorical variables. Using Kaplan–Meier survival curves and the log-rank test, an estimation regarding the survival rates was made. All reported *p*-values are 2-sided. A statistically significant difference was indicated by the values of *p* < 0.05.

## Results

### Patient selection and clinicopathological characteristics

During the study period, 699 patients meeting inclusion criteria were selected, of which 283 patients underwent DP (135 LDP and 148 ODP) and 416 patients underwent PD (128 LPD and 288 OPD). After separate PSM, 86 and 101 patients were matched to the DP and PD groups, respectively (Fig. 1). Details of baseline characteristics of patients undergoing LDP versus ODP and LPD versus OPD are described in Table 1. Patient characteristics such as age, sex, BMI, and ASA were well matched by PSM. Regarding the DP group,there were no differences between the LDP and ODP subgroups in terms of comorbidity, previous abdominal surgery, and the preoperative blood tests for cancer antigen 19–9 (CA19–9) and bilirubin. As for the PD group, 6 (5.9%) and 9 (8.9%) patients in the LPD and OPD subgroups, respectively, previously underwent abdominal laparotomy for other reasons (*p* = 0.42). The LPD and OPD subgroups showed similar preoperative median bilirubin levels (*p* = 0.44) and preoperative median CA 19–9 levels (LPD, 125.7 IU/mL; OPD, 145.7 IU/mL; *p* = 0.95).

**Table 1:** Separate comparison of demographics and clinical characteristics

Variable	LDP (***n*** = 86)	ODP (***n*** = 86)	***p*** value	LPD (***n*** = 101)	OPD (***n*** = 101)	***p*** value
Age (years)^a^	62.7 ± 8.7	62.9 ± 8.8	0.90	62.4 ± 8.2	62.2 ± 8.4	0.87
Sex (Male: Female)	54: 32	54: 32	1.00	67: 34	67: 34	1.00
BMI (kg/m^2^)^a^	22.5 ± 2.5	22.3 ± 2.3	0.53	22.3 ± 2.5	22.5 ± 2.6	0.58
ASA classification (I:II:III)	40: 43: 3	41: 42: 3	0.99	47: 52: 2	45: 54: 2	0.96
Presence of comorbidity (Yes:No)	38: 48	36: 50	0.76	44: 57	46: 55	0.78
Hypertension	26	20		22	31	
Diabetes mellitus	17	15		15	16	
Cardiovascular	2	1		3	3	
Pulmonary	5	4		5	2	
Hepatic	2	1		3	0	
Others	1	1		3	2	
Previous abdominal surgery (%)	7 (8.1%)	8 (9.3%)	0.79	6 (5.9%)	9 (8.9%)	0.42
Preoperative CA19–9 (IU/mL)^b^	108.9 (1.6–3111.0)	113.2 (4.1–3542.0)	0.40	125.7 (4.4–5041.0)	145.7 (1.5–5113.0)	0.95
Preoperative bilirubin (μmol/L)^b^	11.8 (4.6–28.3)	12.5 (5.2–24.3)	0.74	83.5 (4.7–320.8)	94.0 (5.9–390.8)	0.44

a: values were showed as mean (standard deviation) and tested by Student’s *t* test; b: values were showed median (range) and tested by Mann-Whitney *U* test. *BMI* body mass index, *ASA* American Society of Anesthesiologists

### Surgical data and postoperative outcomes

Surgical data and postoperative outcomes are summarised in Table 2. In LDP, one conversion was needed because of adhesions that impeded access to lymphadenectomy. Two other conversions were needed due to bleeding from the splenic vessels. In the LDP group, the mean operative time was significantly shorter (189.1 ± 45.2 vs. 213.3 ± 54.4 min, *p* < 0.01), and median blood loss was significantly lesser (180 [80–600] vs. 220 [120–800] mL, *p* < 0.01) than in the ODP group. Additionally, a lesser number of red blood cell transfusions were required in the LDP group as compared to those in the ODP group (3.5% vs. 11.6%, *p* = 0.04). The median postoperative hospital stay was significantly shorter for LDP patients than for ODP patients (9 [4–34] vs. 13 [7–41] days, *p* < 0.01).

**Table 2:** Separate comparison of surgical data and postoperative outcomes

Variable	LDP (***n*** = 86)	ODP (***n*** = 86)	***p*** value	LPD (***n*** = 101)	OPD (***n*** = 101)	***p*** value
Operative time (min)^a^	189.1 ± 45.2	213.3 ± 54.4	**< 0.01**	416.2 ± 78.8	365.0 ± 81.6	**< 0.01**
Estimated blood loss (mL)^b^	180 (80–600)	220 (120–800)	**< 0.01**	250 (150–900)	300 (180–1000)	**0.04**
RBC transfusion (%)	3 (3.5%)	10 (11.6%)	**0.04**	14 (13.9%)	22 (21.8%)	0.14
Postoperative hospital stay (days)^b^	9 (4–34)	13 (7–42)	**< 0.01**	14 (9–69)	18 (11–52)	**< 0.01**
Overall morbidity (n, %)	8 (9.3%)	14 (16.3%)	0.17	22 (21.8%)	32 (31.7%)	0.11
CR-POPF	5	7		12	16	
DGE (Grade A/ Grade B, C)	0 (0/0)	3 (2/1)		6 (4/2)	10 (5/5)	
PPH (Grade A/ Grade B, C)	0 (0/0)	2 (1/1)		5 (1/4)	6 (3/3)	
Bile leak	0	0		2	3	
Wound infection	0	2		1	1	
Lymphorrhea	1	0		0	2	
Pulmonary complications	2	2		2	5	
Reoperation (%)	0	0	1.00	6 (5.9%)	8 (7.9%)	0.58
Clavien-Dindo classification			0.33			0.42
I-II	4 (4.7%)	5 (5.6%)		10 (9.9%)	16 (15.8%)	
III-IV	4 (4.7%)	9 (10.5%)		11 (10.9%)	15 (14.9%)	
V (90-day mortality)	0 (0%)	0 (0%)		1 (1.0%)	1 (1.0%)	
Adjuvant treatment (%)	61 (70.9%)	57 (66.3%)	0.51	67 (66.3%)	65 (64.4%)	0.77
Time to adjuvant treatment (days)^a^	50 (28–82)	52 (26–88)	0.14	59 (26–98)	60 (26–103)	0.68

a: values were showed as mean (standard deviation) and tested by Student’s *t* test; b: values were showed median (range) and tested by Mann-Whitney *U* test

From amongst the 101 LPD patients, 10 patients (9.9%) required open conversion because of severe adhesion caused by historical abdominal surgery (*n* = 1), intraoperative uncontrollable bleeding from the branches of major vessels (superior mesenteric artery, *n* = 2; gastroduodenal artery, n = 2; portal vein, *n* = 3), and suspicious vascular invasion to achieve safe margins (*n* = 2). LPD showed longer operative time than OPD (416.2 ± 78.8 vs. 365.0 ± 81.6 min, *p* < 0.01). Compared to OPD, LPD showed lesser blood loss (250 [150–900] vs. 300 [180–1000] mL, *p* = 0.04); however, there was no significant difference in the number of red blood cell transfusions required in case of both, LPD and OPD (13.9% vs. 21.8%, *p* = 0.14). The median hospitalization time was longer in the OPD subgroup than in the LPD subgroup (14 [9–69] vs. 18 [11–51] days, *p* < 0.01). Two in-hospital mortalities were noted; each group had one case of POPF and the patients died of multisystem organ failure secondary to sepsis. There were 22 (21.8%) 32 (31.7%) patients in the LPD and OPD groups, respectively, who experienced postoperative complications (21.8% vs. 31.7%, *p* = 0.11). Clinically relevant POPF (12 vs. 16), DGE (6 vs. 10), and pulmonary complications (2 vs. 5) were more frequent in the OPD group; however, these were not significantly different. The severity of morbidity, determined according to the Clavien–Dindo classification, was similar between the LPD and OPD groups (*p* = 0.42).

### Comparison of complete LPD and conversion to open procedure

As shown in Table 3, no significant differences were observed between the complete LPD and conversion groups with regard to age, sex, ASA score, comorbidity, previous abdominal surgery, and preoperative CA19–9 and bilirubin levels; BMI was significantly higher in the conversion group than in the complete LPD group (21.9 [17.4–27.7] vs. 24.2 [20.8–28.3], *p* = 0.02). Regarding surgical outcomes, operative time was similar in both the complete LPD and conversion groups, but blood loss was significantly lesser in the complete LPD group (240 [150–800] vs. 550 [200–900] mL, *p* < 0.01). The median postoperative hospital stay was longer in the conversion group (14 [9–69] vs. 17.5 [14–38] days, *p* < 0.01). Additionally, complete LPD was associated with lower morbidity compared to a conversion to open procedure (18.7% vs. 50%, *p* = 0.04).

**Table 3:** Comparison of complete LPD and conversion to open procedure

Variable	Conversion (***n*** = 10)	Complete (***n*** = 91)	***p*** value
Age (years)	64.5 (52–75)	63 (45–80)	0.55
Sex (Male: Female)	5: 5	62: 29	0.30
BMI (kg/m^2^)	24.2 (20.8–28.3)	21.9 (17.4–27.7)	**0.02**
ASA classification (I:II:III)	5: 5: 0	42: 47: 2	0.88
Presence of comorbidity (Yes:No)	4: 6	40: 51	0.81
Previous abdominal surgery	1 (10%)	5 (5.5%)	0.47
Preoperative CA19–9 (IU/mL)	177.5 (40.4–1790.0)	120.4 (4.4–5041.0)	0.47
Preoperative bilirubin (μmol/L)	64.0 (8.3–320.8)	87.4 (4.7–303.1)	0.57
Operative time (min)	455 (320–490)	400 (270–680)	0.39
Estimated blood loss (mL)	550 (200–900)	240 (150–800)	**< 0.01**
Postoperative hospital stay (days)	17.5 (14–38)	14 (9–69)	**< 0.01**
Overall morbidity (n, %)	5 (50%)	17 (18.7%)	**0.04**
Reoperation (%)	1 (10%)	5 (5.5%)	0.47
Tumor size	3.5 (1.5–5.0)	3.0 (1.5–5.4)	0.56
Radical R0 resection (%)	8 (80%)	86 (94.5%)	0.14
Retrieved lymph node	20 (16–30)	22 (13–54)	0.47
Vascular invasion (%)	3 (30%)	14 (15.4%)	0.37
Perineural invasion (%)	6 (60%)	38 (41.8%)	0.33

Note: all continuous data were showed as median (range) and compared by Mann-Whitney *U* test due to abnormal distribution in conversion group

### Pathological examination and oncological outcomes

The pathological examination outcomes are shown in Table 4. Tumour size, cell differentiation, pT stage, and pN stage were similar in the LDP and ODP subgroups. The LDP subgroup was associated with a significantly higher number of harvested LNs than the ODP subgroup (14.4 ± 5.2 vs. 12.7 ± 5.0, *p* = 0.03), whereas the radical R0 resection rates, vascular, and perineural invasion were similar between the LDP and ODP subgroups. After PSM, pathological examination revealed that tumour size, pT-stage, and pN-stage were well matched between the LPD and OPD subgroups. The LPD subgroup tended to have more harvested LNs than the OPD subgroup (22.6 ± 6.5 vs. 21.0 ± 6.2, *p* = 0.07). The R0 rates and vascular perineural invasion were similar between the LPD and OPD subgroups.

**Table 4:** Separate comparison of pathological examination

Variable	LDP (***n*** = 86)	ODP (***n*** = 86)	***p*** value	LPD (***n*** = 101)	OPD (***n*** = 101)	***p*** value
Tumor size^a^	41. ± 1.5	4.2 ± 1.4	0.97	3.0 ± 0.9	3.1 ± 1.0	0.60
Differentiation			0.84			0.56
Well	34	36		33	39	
Moderate	31	27		40	40	
Poor	21	23		28	22	
Radical R0 resection (%)	83 (96.5%)	78 (90.7%)	0.12	94 (93.1%)	90 (89.1%)	0.32
Retrieved lymph node^a^	14.4 ± 5.2	12.7 ± 5.0	**0.03**	22.6 ± 6.5	21.0 ± 6.2	0.07
Vascular invasion (%)	17 (19.8%)	18 (20.9%)	0.85	17 (16.8%)	20 (19.8%)	0.59
Perineural invasion (%)	41 (47.7%)	38 (44.2%)	0.65	44 (43.6%)	45 (44.6%)	0.89
Pathologic T stage			0.93			0.97
T1	3	4		14	15	
T2	44	45		72	72	
T3	39	37		15	14	
Pathologic N stage			0.66			0.60
N0	42	46		50	53	
N1	35	29		44	38	
N2	9	11		7	10	

a: values were showed as mean (standard deviation) and tested by Student’s *t* test

**Fig. 2:**
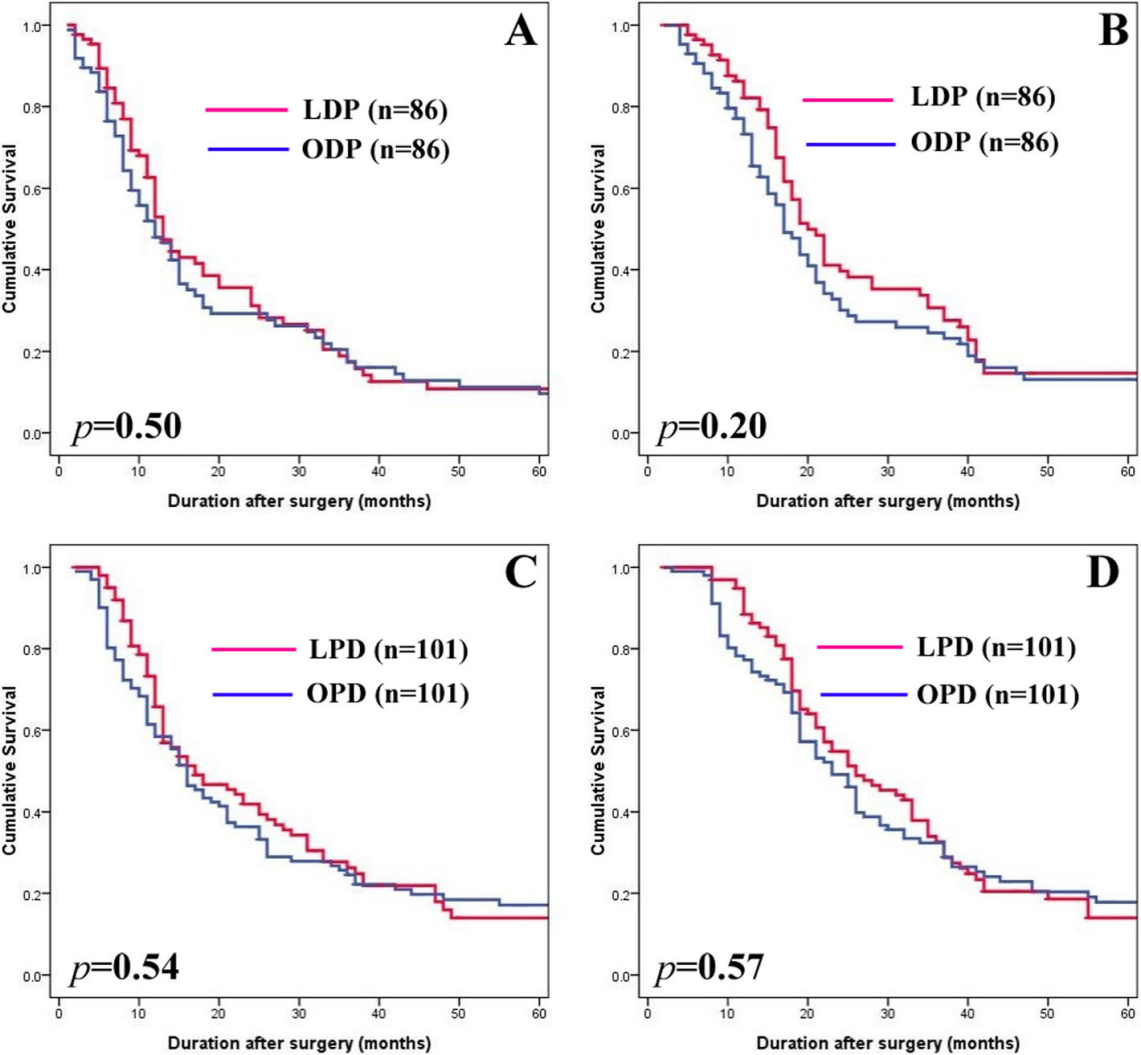
Kaplan-Meier survival curves. **a** Cumulative DFS between LDP and ODP. **b** Cumulative OS between LDP and ODP. **c** Cumulative DFS between LPD and OPD. **d** Cumulative OS between LPD and OPD

The median follow-up times were 17 (2–120) months and 15.5 (3–108) months for the LDP and ODP subgroup, respectively. Recurrence occurred in 65 patients (75.6%) in the LDP subgroup (22 locoregional, 25 extrapancreatic, and 18 combined locoregional/extrapancreatic recurrences) and 70 patients (81.4%) in the ODP subgroup (18 locoregional, 26 extrapancreatic, and 26 multiple recurrences). There were no statistical differences in DFS and OS between the LDP and ODP subgroups (Fig. 2a, b).

The median follow-up times were 22 (2–63) months and 33 (3–124) months for the LPD and OPD subgroups, respectively. Recurrence occurred in 73 patients (72.3%) in the LPD subgroup (18 locoregional, 36 extrapancreatic, and 19 combined locoregional/extrapancreatic recurrences) and 81 patients (80.2%) in the OPD subgroup (20 locoregional, 33 extrapancreatic, and 28 multiple recurrences). There were also no statistical differences in recurrence patterns, DFS, and OS between the LPD and OPD subgroups (Fig. 2c, d).

## Discussion

The use of LDP for PDAC is still debatable, although LDP provides a significant alternative for benign or low-grade tumour on pancreatic bodies and tails [5, 8]. Two surveys suggested that 19–31% of surgeons expected LDP to be inferior to ODP in PDAC treatment [26, 27]. On the other hand, approaching PDs laparoscopically for diseases on pancreatic heads was less frequent owing to the intricacy of the dissection and the complexity of the pancreatoenteric and biliodigestive anastomoses [6, 10]. Moreover, LPD for PDAC is still in its infancy because of concerns about the safety and oncological efficacy [10]. Therefore, it is necessary to separately evaluate the efficacy of LDP and LPD for PDAC treatment. This study suggests that both the procedures are technically feasible and safe, and both consistently exhibit clear benefits (less blood loss and short hospital stay). LDP and LPD have no obvious advantages in decreasing postoperative morbidity. LDP appears advantageous for the retrieval of lymph nodes. Additionally, long-term survival outcomes were closely matched between laparoscopic and open surgery.

The feasibility of LPD was the major concern in the adoption of this surgical technique, as a high conversion rate was reported, especially in the initial period. In ITT analysis, the conversion rates were only 3.5% (3/86) for LDP cases but 10 (9.9%) conversions for LPD cases. Two recently published randomised clinical trials (RCTs) reported that the conversion rates for LPD were more than 20% [28, 29]. A high conversion rate indicates that LPD for PDAC remains a challenging procedure. The most common reasons for conversions were uncontrollable haemorrhages or suspected vessel involvement, which was similar to other publications on LPD for PDAC treatment [30, 31]. Nickel et al. reviewed six studies focusing on the learning curve of LPD and revealed that the volume to reach a technical competency ranged from 10 to 60 cases depending on the surgeon’s expertise [32]. Our conversion cases were also mainly in the initial period, which may partially explain the high conversion rate of the RCTs, although surgeons had an experience of handling at least 10 cases of LPD before anticipating the RCTs. In our centre, the surgical team was competent in performing advanced procedures, including reconstruction of the gastrointestinal tract, intracorporeal suture, and emergency haemostasis. We believe that training together in a relatively constant surgical team contributes to better surgical outcomes, especially in emergencies during LPD, which may turn into conversion. Interestingly, conversion was found to be associated with higher patient BMI. The advantage of less blood loss in LPD was more obvious when data on conversion were omitted, and the difference in overall morbidity between LPD and OPD was statistically significant (17/91 [18.7%] vs. 32/101 [31.7%], *p* = 0.04). We believe that part of the reason for high conversion is that PDAC frequently induces substantial pancreatic inflammation in the pancreatic remnant, which is difficult to resect due to pronounced adhesions or infiltrations to the surrounding tissues or vessels. Portomesenteric vein (PV) involvement is a common clinical finding in PDACs, but it is difficult to diagnose prior to surgery [33]. However, researchers strongly recommend that the PV should be resected, once detected as contributing to a tumour by surgeons with considerable proficiency in vascular resection and reconstruction [34, 35]. As for LPD, approaching appropriate cases without vessel involvement or severe adhesions laparoscopically, avoiding obese or overweight patients in the learning curve would be helpful in reducing conversion [28].

According to our data, LDP had a reduced time of operation, but an extended operative time in LPD in comparison with their open counterparts. The former is largely attributed to the simplicity of LDP, and fast management of the trocar incision could reduce the time required for laparoscopic resection. Comparatively, LPD poses a tougher challenge for surgeons because it involves not only complex dissection but also complex gastrointestinal, pancreatic, and biliary anastomoses, all of which present a technical challenge. Our initial LPD for PDAC lasted for nearly 600 min [16]. Currently, this can be completed in approximately 300–350 min [15]. Kendrick et al., in one of the largest single series studies, described initial LPD duration to be 460 min, which improved to 320 min after about 50 cases [36]. Stauffer et al. suggested an average operation time of 518 min, which was clearly more than that required for an open surgery (140 min) [31]. The learning curve can be overcome in high-volume centres, with average LPD operative times reduced to lower than 400 min [37]. Nevertheless because of tumour biology and the progression of the disease, LPD for PDAC treatments is not regarded as a common option; as a result, it is challenging to address the related learning curve [6]. A longer operation time was related to the worse perioperative outcomes following pancreatic resections, according to a research conducted under the American College of Surgeons National Surgical Quality Improvement Program [38]. Therefore, we believe that a long duration is a definite disadvantage of LPD for PDAC treatment.

We found that both LDP and LPD showed a trend of less overall morbidity without statistical significance in contrast to ODP and OPD (DP: 9.3% vs. 16.3%, *p* = 0.17; PD: 21.8% vs. 31.7%, *p* = 0.11). POPFs are commonly viewed as the most ominous of complications after pancreatic resection, and the most effective management for the pancreatic stump is still under debate, although various surgical procedures of pancreatic stump management after DP and anastomosis after PD have been devised to prevent POPFs in conventional open surgery [11, 39]. As laparoscopic surgical instruments are developed and the operative experience is accumulated, it is possible to do open surgery performed laparoscopically using the same reported methods [6, 11]. DGE is not life threatening, but can have significant consequences such as patient discomfort, prolonged hospital stays, diminished nutritional status, and delays in the initiation of adjuvant therapy [40, 41]. The pathogenesis of DGE is multifactorial. Given improved access and visualisation, as well as the meticulous attention, a laparoscopic approach could theoretically reduce DGE because of the following reasons [42, 43]: 1) it can mitigate the impact on the organs and peritoneum, leading to less seroperitoneum which helps alleviate gastric dysrhythmias, 2) it can ameliorate pyloric or antral ischaemia as a result of reserved small vessels, and 3) it can mitigate pylorospasms secondary to denervation of the stomach and duodenum or jejunum. Additionally, open procedures are reported to portend a higher risk of pleural effusions, pulmonary infections, and atelectasis than minimally invasive ones [44, 45].

Before the widespread application of a new surgical approach, oncologic safety and effectiveness should be verified. The long-term survival outcomes of MISs for common malignancies have conflicting results [46–48], leading to a constant controversy over MIS for cancer treatments. Nassour et al. used the NCDB database to compare the long-term oncologic outcomes of LPD and LDP to open surgery in patients with PDAC, and found that MIS was associated with similar long-term survival for PD, and improved survival for DP [49]. Our study revealed that R0 resection of LDP and LPD are similar to open surgery. It is worth noting that preoperative serum levels of CA 19–9 predict resectability and survival [50]. Patients with CA19–9 levels > 4000 U/mL had a resection rate of 38% [50]. In the present study, a majority of patients had mildly elevated CA199 levels, except those combined with diseases of the biliary tract, while preoperative examination showed no signs of metastasis. Additionally, our data showed that LNs retrieved in LPD patients were not inferior to those of OPD (22.6 ± 6.5 vs. 21.0 ± 6.2, *p* = 0.07), and the LNs harvest of LDP was superior to ODP (14.4 ± 5.2 vs. 12.7 ± 5.0, *p* = 0.03). Noticeably, the DIPLOMA research revealed that LDP was linked to a higher R0 resection rate (67% vs. 58%) and a smaller number of LNs (14 vs.22) [51]. However, this pan-European PSM study discovered that lower LN retrieval with LDP does not make a noticeable difference to the average OS.(28 vs. 31 months) [51]. In general, it was revealed by studies, including meta-analyses, that the long-term outcomes of LDP for PDAC are promising [52–55]. As for LPD, a single-centre study conducted by Asbun and Stauffer reported similar long-term survival rates at 1, 2, 3, 4, and 5 years for OPD (68, 40, 24, 17, and 15%) and LPD (67, 43, 43, 38, and 32%), respectively [31]. Kuesters et al. conducted a series of LPD procedures and reported a similar 5-year survival rate between LPD (20%) and OPD (14%) for PDACs [56]. Some publications have demonstrated that LPD has a positive effect on long-term oncologic outcomes in patients with PDAC [30, 57]. It was hypothesised that the enhanced recovery following laparoscopic surgery was conducive to activating multimodality therapies in advance, thus improving survival [30]. Nevertheless, according to a retrospective analysis of the NCDB, MIS failed to improve the use or initiation of adjuvant chemotherapy for patients with PDAC [58]. Moreover, the survival effect of the activation time of adjuvant chemotherapy in patients with resected PDAC remains unknown, as studies have indicated contradictory outcomes [59, 60]. A technically similar oncologic resection is worth performing, irrespective of the open or laparoscopic approach if the principles of radical resection are complied with. Accordingly, Lee et al. compared resected PDAC in a study, which included both laparoscopic and open cases, and found that according to the Yonsei criteria (a preoperative CT-based determined method) can predict excellent short-term and long-term oncologic outcomes [61]. In other words, if surgery follows the oncologic principle, the oncologic impact is not influenced by differences in the surgical approach [61].

The limitations of this study include its retrospective design, small sample size, the absence of randomisation, and a short follow-up period. Although PSM was performed to balance the covariates, thus reducing selection bias, other factors cannot be ignored. In the PD arm, the follow-up period of LPD was shorter than that of OPD since LPD was initially conducted in late 2012. Additionally, ODP has been largely performed in early years, in contrast to LDP. This can give rise to bias, taking postoperative management as an example. Recently, surgeons are unavoidably influenced by the concept of enhanced recovery after surgery, which includes early mobilisation, oral feeding, midthoracic epidural analgesia, and premature removal of abdominal drain; it can reduce the length of hospitalisation and recovery in postoperative management [62], causing bias in favour of the LDP and LPD group. The limited follow-up period of LDP and LPD is insufficient to provide enough information on long-term outcomes. Additionally, the sample size hinders the effort to arrive at reliable conclusions, especially regarding several variables distinct between the groups, but with no significance revealed. Considering a standard approach for borderline resectable or locally advanced PDAC [63], neoadjuvant chemotherapy for resectable PDAC was applied in some high-volume centres but was not applied in our centre before 2017, so oncological outcomes of pancreatectomy for PDAC patients after neoadjuvant chemotherapy still need further analysis.

## Conclusions

Laparoscopic pancreatectomy was safe and effective for the treatment of PDAC; LDP and LPD were associated with less blood loss and shorter hospital stay. However, the short-term surgical advantage of LPD is not as obvious as those of LDP, mainly due to the surgical conversions. The oncological outcomes of LDP and LPD were not inferior to those of traditional open procedures for the treatment of PDAC. Future studies may consider longer follow-up periods and larger patient samples to validate our findings.

## Data Availability

The datasets generated and/or analyzed during the current study are not publicly available due to data privacy according to the license for the current study, but are available from the corresponding author on reasonable request.

## Abbreviations

PDAC: Pancreatic ductal adenocarcinoma
LPR: Laparoscopic pancreatic resection
LPD: Laparoscopic pancreaticoduodenectomy
OPD: Open pancreaticoduodenectomy
DP: Distal pancreatectomy
MIS: Minimally invasive surgery
PSM: Propensity score matching
SD: Standard deviation
LN: Lymph node
ITT: Intention-to-treat
POPF: Postoperative pancreatic fistula
CR-POPF: Clinically relevant POPF
DFS: Disease-free survival
OS: Overall survival
PJ: Pancreaticojejunostomy
RCT: Randomised clinical trial
PV: Portomesenteric vein
DGE: Delayed gastric emptying
BMI: Body mass index
ASA: American Society of Anesthesiologists
RBC: Red blood cell
